# Comparative Evaluation of Fracture Resistance of Endodontically Treated Teeth Instrumented With K3XF Rotary Files Using Different Tapers

**DOI:** 10.7759/cureus.34247

**Published:** 2023-01-26

**Authors:** Pradeep Bapna, Afzal Ali, Saleem D Makandar, Nik Rozainah Nik Abdul Ghani, Sandeep Metgud

**Affiliations:** 1 Conservative Dentistry and Endodontics, Pacific Dental College and Hospital, Udaipur, IND; 2 Conservative Dentistry and Endodontics, School of Dental Sciences, Health Campus Universiti Sains Malaysia, Kubang Kerian, MYS

**Keywords:** different tapers, endodontically treated teeth, fracture resistance, k3xf file system, taper rotary files

## Abstract

Aim: To compare the effect of different tapers of the K3XF file system on the fracture resistance of endodontically treated mandibular premolars obturated with a three-dimensional (3-D) obturation system.

Methodology: For the study, 80 freshly extracted human mandibular premolars with single well-developed roots without any curvatures were taken and the tooth roots were wrapped in a single layer of aluminum foil, and they were placed vertically in a plastic mold filled with self-curing acrylic resin. The access was opened, and working lengths were determined. The canals were instrumented keeping an apical size of #30 by different taper rotary files: Group 1: un-instrumented (control group), Group 2: 30/.04, Group 3: 30/.06, Group 4: 30/.08 K3XF file system, and teeth were obturated using a 3-D obturation system, and access cavities were filled using composite. Both experimental and control groups were subjected to fracture load using a conical steel tip (0.5mm) attached to a universal testing machine to record force applied in newton until root fracture.

Results: Root canal instrumented groups showed lower fracture resistance than the uninstrumented group.

Conclusion: Hence it could be concluded that endodontic instrumentation with increased taper rotary instruments caused a decrease in fracture resistance of the teeth, and biomechanical preparation of root canal system with rotary or reciprocating instruments caused a significant decrease in fracture resistance of endodontically treated teeth (ETT), thereby decreasing their prognosis and long-term survival.

## Introduction

Endodontic therapy consists of steps that aim to remove the contaminated pulp and dentin from the root canal and repair the periapical tissues that guarantee tooth functioning [1]. The shaping, cleaning, and disinfection would allow for a three-dimensional obturation (3D) of the root canal system from a biomechanical standpoint [2]. Extreme tooth structure loss due to trauma or caries, access cavity preparation and design, dryness of dentin, instrumentation with rotary files, excessive pressure during filling procedures, detrimental effects of irrigation solutions, and preparation for intra-radicular post space are all factors that affect the strength of root canal treated teeth and has been shown in studies to increase root fracture susceptibility [3-7]. In terms of clinical outcomes, these fractures may result in a poor prognosis of endodontic treatment and lower the long-term survival rate of the tooth.

Inspired by the minimally invasive dentistry concept, a conservative access cavity (CAC) has been designed which minimizes the removal of pulp chamber roof and peri-cervical dentin (PCD) [8]. The dentin near the alveolar crest is known as PCD. Although the coronal third of the clinical crown and the apex of the root can be amputated and prosthetically replaced, the dentin near the alveolar crest cannot. This vital zone, nearly 4 mm above the crestal bone and extending 4 mm apical to the crestal bone, is imperative for three reasons: (a) fracturing, (b) ferrule, and (c) proximity of the dentin tubule orifice from inside to out [8]. According to Sabeti et al. and Taha Ozyurek et al., no significant differences have been reported in fracture resistance of the tooth prepared using CAC and traditional access cavities [9,10].

Cervical preflaring reduces the incidence of operative accidents and permits more precise calculation of working length and apical diameter. Traditionally, Gates Glidden (GG) has been employed for this purpose. However, it offers certain limitations like the hourglass appearance at the cervical area of root canals. Nowadays, Nickel-Titanium (Ni-Ti) rotary orifice openers are used for cervical flaring. The GG drills have been reported to cause more crack formation, thereby reducing the fracture resistance of root-filled teeth compared with Ni-TI orifice openers [11].

Multiple research has been carried out over the last decade in comparing the fracture resistance of teeth instrumented with rotary Ni-Ti instruments and traditional hand, with the conclusion that increased taper advocated by Ni-Ti rotary files does not weaken roots any more than traditional stainless steel hand files, and may even increase the fracture resistance of teeth [12]. Another factor that could influence vertical root fracture (VRF) predisposition is the horizontal dimension of the prepared canal. Excessive tapering may result in excessive dentin removal and root weakening, increasing the root's vulnerability to fracture [13]. The K3 rotary Ni-Ti instrument was used in the present study, an example of third-generation rotary Ni-Ti instruments developed and marketed by Sybron Endo (Orange, CA, USA). For cutting parameters effectiveness, the K3 has an irregular design with a mildly positive rake angle. The variable helical flute angle made it easier to remove dentin chips from the working area and transport them to the orifice. It comes in a variety of tip sizes (#15 to #60) and taper (0.02-0.12). Because there is limited research on the fracture resistance of teeth instrumented with the Sybron Endo K3XF rotary file system, the current study aims to assess the fracture resistance of endodontically treated teeth (ETT) using different taper rotary instruments. In this study, the null hypothesis, there was no statistically significant difference between the groups.

## Materials and methods

The sample size was calculated in G-power software. Eighty human permanent single-rooted mandibular first and second premolars with fully formed apex extracted for orthodontic purposes were collected from the Department of Oral and Maxillofacial Surgery, after prior ethical approval from the ethical committee (PDCH/19/EC/164). Teeth were examined under a stereomicroscope at a magnification of 45X to check if any crack/craze lines were present, the x-rays were taken to check for calcification, caries, fractured old restoration, resorption defects, and root canal treated teeth, any tooth with these findings was excluded from the study. To ensure homogeneity, teeth with approximately the same weights and same lengths were chosen.

The samples were disinfected by immersing them in 10% formalin solution for seven days and then cleaned using an ultrasonic scalar. Teeth were stored for three months in 0.9% physiologic saline before further use. To prevent the flow of acrylic resin, the roots of each sample were wrapped in a single layer of aluminum foil and then placed vertically in a plastic mold packed up to the level of the cervical margin of the tooth (CEJ) with self-cure acrylic resin. All the specimens were radiographed in mesiodistal and buccolingual directions to ensure vertical positioning in their artificial socket. Just after the polymerization of acrylic resin, the aluminum foil was removed, and roots were coated with light body addition silicone impression material to mimic an artificial periodontal ligament space and placed back into the resin block.

**Figure 1:**
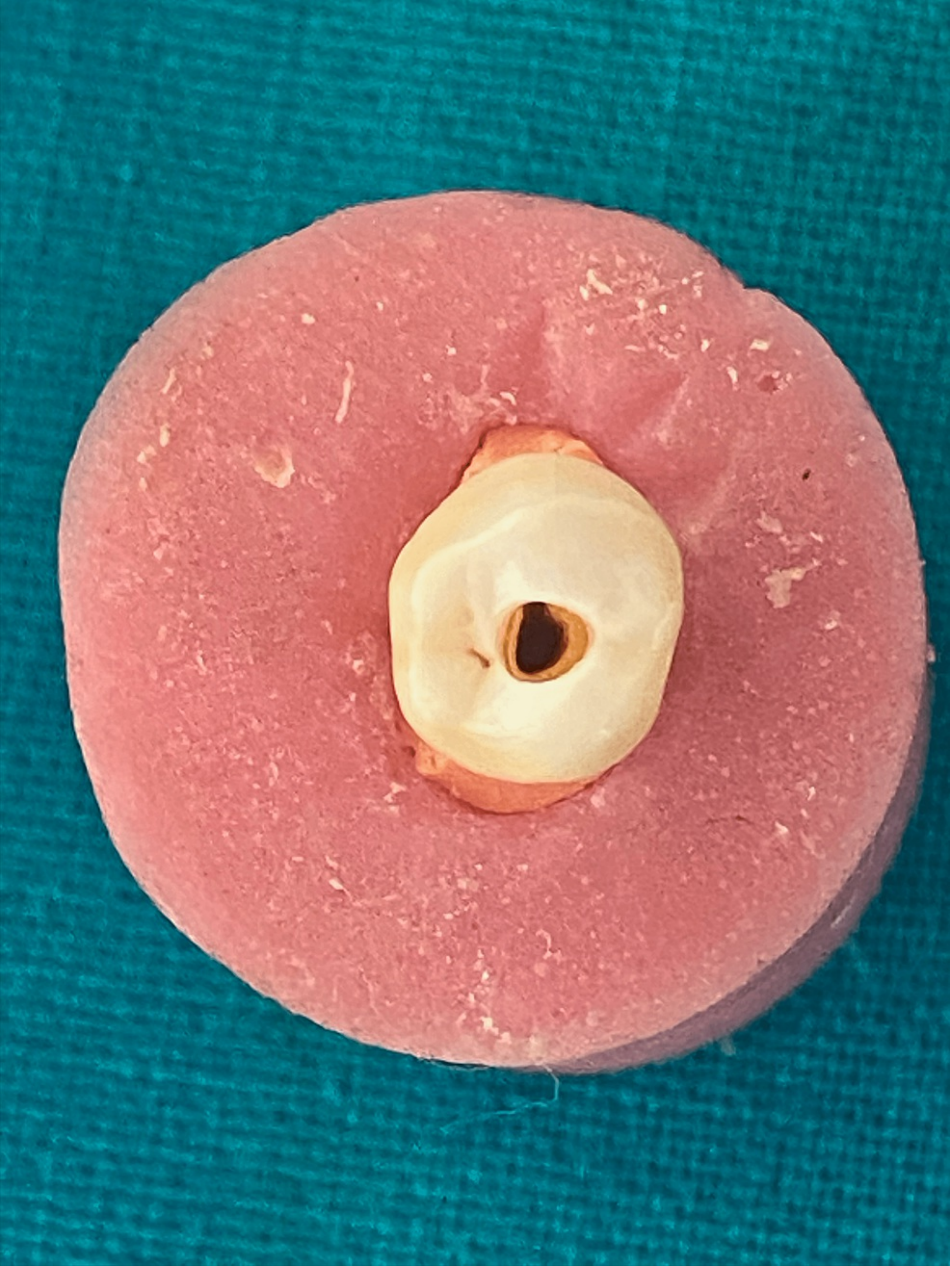
Minimal access cavity design

A minimal invasive access cavity was prepared using #4 round bur (Dentsply Maillefer, Switzerland) in such a way that the preparation started usually at the central fissure of the occlusal surface, extended towards pulp with smoothly convergent walls, and canals were located (Figure 1). The patency of the canal was established using a #15 k file (Dentsply Maillefer, Switzerland) followed by working length determination using radiographs. Eighty specimens were distributed randomly and equally divided into four groups (n=20).

Except for the control group, the remaining samples were then instrumented, keeping the apical size of #30 by different taper rotary instruments using the crown-down technique. The groups were as followed, Group 1: samples left uninstrumented, Group 2: instrumented up to 30/.04 taper of K3 rotary file system (Sybron Endo, Orange, CA, USA), Group 3: instrumented up to 30/.06 taper of K3 rotary file system (Sybron Endo, Orange, CA, USA), Group 4: instrumented to 30/.08 taper of K3 rotary file system (Sybron Endo, Orange, CA, USA).

The canals were irrigated with 3% NaOCl and distilled water during instrumentation (Prime dental products, Mumbai, India). Following instrumentation, a final flush of 5 ml 17% EDTA (Pyrax Polymar, India) and 5 ml 2.5% NaOCl was applied for 1 minute, later by a last rinse of 5 ml distilled water for 1 minute using a 27G side-vented needle. The canals were desiccated with paper points and filled three-dimensionally using Fast Pack and Fast-Fill Obturation system (Channgzhou Saifary Medical Technology Co. Ltd., Jiangsu Province, China) and Sealapex sealer (Kerr, Romulus, MI). The gutta-percha was removed from the orifices, cleaned the access with normal saline. The access cavity was etched with 37% phosphoric acid etchant for 30 seconds and thoroughly washed using water spray with a three-way syringe then bonding agent (Coltene-Whaledent, Switzerland), was applied using a micro brush with agitation, and the air was spread slowly onto the bonding agent to get homogenous distribution then cured with LED curing light (SmartLight Pro Dentsply Sirona) for 20 seconds. The access cavity was restored using composite resin in increments according to the manufacturer's instructions (Coltene-Whaledent, Switzerland).

**Figure 2:**
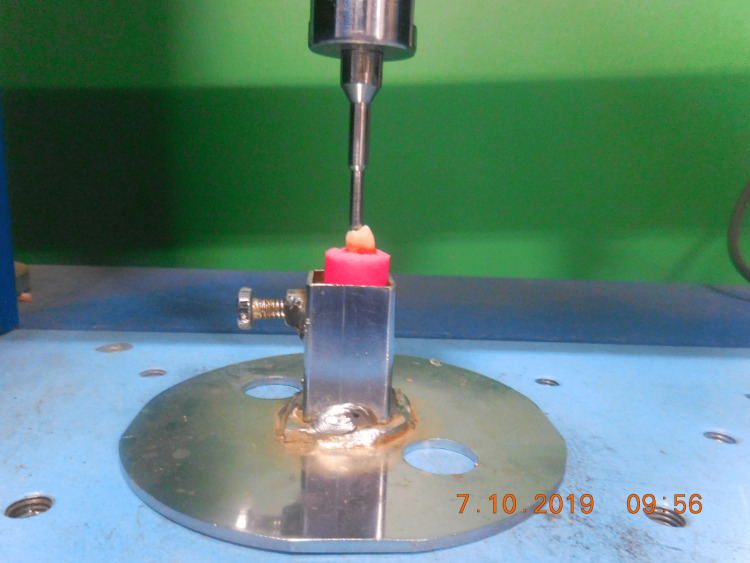
The placement of the needle and assembly on a universal testing machine

The samples were mounted on a universal testing machine, which used a steel conical tip with a diameter of 0.5mm and a speed of 1.25mm/min, allied with the access cavity's center for each specimen (Figure 2). The load required to cause root fracture was measured in newtons and applied at a crosshead speed until root fracture occurred. For each group, the mean values and standard deviation were calculated. All the data were then statistically evaluated using the One-way Analysis of Variance (ANOVA) test and the Post-hoc Tukey's test. The Statistical Package for the Social Sciences software (SPSS) version 20 was used to analyze the data (SPSS Inc., Armonk, NY). P < 0.05 was chosen as the statistical significance level.

## Results

The fracture resistance was found to be significantly high in Group 1 (control group) (518.1N) followed by Group 2 (314.6N), Group 3 (276.7N), and Group 4 (154N) (Table 1). In all the groups a statistically significant difference was observed. In instrumented groups, lower fracture resistance was observed as compared to uninstrumented (control group). Thus, endodontic instrumentation with increased taper rotary instruments caused a reduction in fracture resistance of teeth.

**Table 1:** The fracture resistance of teeth in four groups was compared using different tapering rotary instruments Test applied: One-way Analysis of Variance (ANOVA) test; * p-value <0.05 statistically significant

Groups	N	Mean fracture resistance (in Newton)	Standard deviation	p-value
Group 1 (Control)	20	518.1	13.52	<0.01*
Group2 (30/04%)	20	314.6	19.41	
Group 3 (30/06%)	20	276.7	19.79	
Group 4 (30/08%)	20	154	17.77	
Total	80	316.03	132.75	

Because there was a statistically significant difference between all groups, the null hypothesis was rejected. The difference in fracture resistance between Groups 1 (control group) and Group 4 was significantly higher (30/8%) compared to other groups' mean differences (Table 2). This shows that increasing the taper of the root canal weakens the tooth.

**Table 2:** Comparison of mean differences in fracture resistance of teeth between groups using different tapering instruments Test applied: Post-hoc Tukey test; * p-value <0.05 statistically significant

Comparisons	Mean differences	p-value
Group 1 vs Group 2	203.50	<0.01*
Group 1 vs Group 3	241.40	<0.01*
Group 1 vs Group 4	363.4	<0.01*
Group 2 vs Group 3	37.90	<0.01*
Group 2 vs Group 4	159.90	<0.01*
Group 3 vs Group 4	122.0	<0.01*

**Figure 3:**
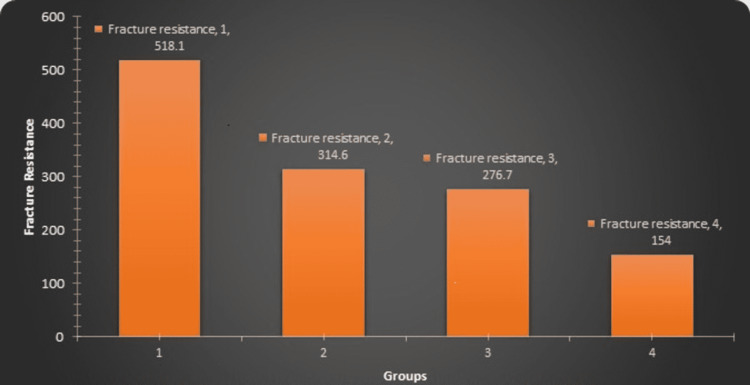
The fracture resistance of teeth in four groups was compared using different tapering rotary instruments

The fracture resistance of teeth when instrumented with various taper rotary instruments is depicted in Figure 3. The fracture resistance of the teeth decreased as the taper of the rotary instrument increased.

## Discussion

The ultimate objective of root canal therapy is the eradication of contaminated dentin, necrotic tissue, and microbes from the root canal space [14]. Endodontic therapy is carried out by removal of the diseased pulp, cleaning, and disinfection of root canal space, and obtaining a hermetic seal by obturation [15]. Then a definitive post-endodontic restoration is planned for the long-term prognosis of such teeth, as ETT is more prone to fracture under the masticatory load.

Occlusal stresses associated with normal mastication and paranormal function (bruxing, clenching) can cause compression and tension in the periodontal ligament, enabling the teeth to move. The natural periodontal ligament is preserved with ETT, enabling physiologic movement, reacting to, and adjusting to functional occlusal forces to enable greater occlusal contact during biting [16]. VRF is a serious clinical issue because it signifies a poor prognosis for the teeth involved. VRF is caused by excessive canal shaping, excessive restorative procedures, excessive pressure during obturation, excessive width and length of post space in relation to the tooth's anatomy and morphology, and inappropriate choice of the tooth for a bridge abutment [13].

The instrumented groups in the current study had lower fracture resistance than the control group (p < 0.01) (Table 2). This loss of dentin can be attributed to the use of various tapers (4%, 6%, 8%) of K3XF rotary instruments and surface treatment of the root canals with the irrigants. The results of our study are in agreement with the studies conducted by Sabeti et al., Krikeli et al., and Rundquist et al., who noticed a decrease in fracture resistance with the use of increased taper rotary instruments. The extraction and storage time may increase the brittleness of the tooth samples. Hence in the present study, the extracted teeth were collected and stored for three months in 0.9% physiologic saline before use [10,17,18]. However, Hegde et al. [12] concluded that an increase in taper did not affect the fracture resistance of ETT.

When the tested groups were compared to each other (Table 1), a statistically significant difference was reported between them. However, the mean difference in Group 2 and Group 3 was significantly less (37.9N) than the mean differences among other groups. This proved that there was a statistically significant difference in the fracture resistance of 4% and 6% taper, hence the null hypothesis was rejected. Amongst the instrumented groups, Group 4 exhibited the lowest, and Group 2 exhibited the highest fracture resistance. This can be attributed to the increase in the taper of instruments that leads to more dentin removal. Hence, it can be concluded that using greater taper files like 8% caused a considerable decrease in fracture resistance of ETT and hence must be avoided.

Furthermore, the negative influence of root canal irrigants may have weakened the root dentin. Moreover, the method used for testing fracture load was static load in this study, whereas, in the intra-oral condition, a dynamic load is applicable. Further investigations into other types of Ni-Ti instrument teeth may give further insights into the effects of different rotary Ni-Ti instruments on the fracture strength of teeth and susceptibility to VRF [19].

## Conclusions

Within the limitations of the current study, it is possible to conclude that biomechanical preparation of the root canal system with rotary or reciprocating instruments resulted in a significant reduction in fracture resistance of root canal-treated tooth, thereby decreasing its prognosis and long-term survival. Fracture resistance of teeth instrumented with 4% or 6% files shows significant differences from each other and can be used cautiously. The use of greater taper instruments caused a marked reduction in ETT fracture resistance and questioned its usage.
